# Proximate composition, functional, and pasting properties of wheat and groundnut protein concentrate flour blends

**DOI:** 10.1002/fsn3.670

**Published:** 2018-05-17

**Authors:** Ocheme Boniface Ocheme, Olajide Emmanuel Adedeji, Chiemela Enyinnaya Chinma, Caleb Maina Yakubu, Ugochukwu Happiness Ajibo

**Affiliations:** ^1^ Department of Food Science and Technology Federal University of Technology Minna Nigeria; ^2^ Department of Food Science and Technology Federal University Wukari Nigeria

**Keywords:** composite flour, groundnut, protein concentrate, wheat

## Abstract

This study investigated the effect of groundnut protein concentrate inclusion on the quality of wheat flour. Wheat and groundnut concentrate flours were blended (%, w/w) at ratios 100:0, 95:5, 90:10, 85:15, and 80:20, with 100% wheat flour serving as the control sample. Subsequently, proximate composition, functional, and pasting properties of blends were determined using established methods. Protein content of the concentrate was 72.80%. Significant (*p* < .05) increase in protein content of the flour blends was recorded with increasing concentration of groundnut protein concentrate and decreasing concentration of wheat flour. Highest protein content of 28.87% was recorded in blend with 20% groundnut protein concentrate. Fat, ash, and crude fiber contents ranged from 1.93% to 8.84%, 0.70% to 1.13%, and 0.84% to 1.23%, respectively. Carbohydrate decreased significantly (*p* < .05) with increasing concentration of groundnut protein concentrate. Bulk density and swelling capacity of the flour blends reduced significantly (*p* < .05) with increasing level of groundnut protein concentrate flour while solubility index, water absorption, emulsion, and foaming capacities increased. Peak time, peak, trough, breakdown, final, and setback viscosities of the flour blends reduced with groundnut protein concentrate inclusion while the pasting temperature reduced. Overall, wheat/groundnut protein concentrate flour blends showed good functional and pasting properties.

## 1 INTRODUCTION

Wheat (*Triticum aestivum L*.) flour is the major raw material employed in many baking processes (Okpala & Egwu, 2015). The suitability of wheat in the production of many confectionary products is as a result of good intrinsic viscoelastic properties of the wheat protein (gluten; Ibitoye, Afolabi, Otegnayo, & Akintola, 2013). It also contains 78.10% carbohydrates, 14.7% protein, 2.10% fat, 2.10% minerals, and significant amounts of vitamins such as thiamine and vitamin B (Okpala & Egwu, 2015).

According to Nwanekezi (2013), the importation of wheat in Nigeria engulfs a whooping sum of 635 billion Naira annually. In addition, wheat has been reported to be low in essential nutrient such as lysine (Olapade & Oluwole, 2013) and has also been implicated for the incidence of celiac disease: a systemic immune‐mediated disorder caused by the ingestion of gluten‐containing grains (Lionetti, Gatti, Pulvirenti, & Catassi, 2015). As a result of these, several efforts were made to neutralize the problems associated with wheat consumption through the use of locally available plant products (Ocheme, Oloyede, & Mahmud, 2010; Ade, Ingbian, & Abu, 2012; Adegunwa, Bakare, & Akinola, 2012; Olapade & Oluwole, 2013; Ugwuona & Suwaba, 2013; Ibitoye et al., 2013). Groundnut (*Arachis hypogea*) like many other nuts has also been used for the improvement of wheat‐based confections (Ajanaku, Ajanaku, Edoboh‐Osoh, & Nwinyi, 2012; Bindhya & Kochhar, 2013; Asimah, Kpodo, Adzinyo, & Dzah, 2016).

The use of legumes or nuts for fortification or enrichment of cereal‐based foods is not without its challenges. First, the beany flavor of many legumes often results in low acceptability (Mune, Minka, & Mboma, 2013). Second, there is the problem of poor digestibility of leguminous crops owing to the presence of oligosaccharides which are not easily digested (Gayol, Pramparo, Nepote, Fernendez, & Grosso, 2013). Consequently, proteins in legumes/nuts are of poor quality and low biological value (Garba & Kaur, 2014). Furthermore, flours from many leguminous crops have been reported to have poor functional properties (Ajanaku et al., 2012). Consequently, several research efforts have been made to concentrate or isolate proteins from legumes/nuts for improved protein functionality, digestibility, and bioavailability (Ogunbusola, Fagbemi, & Oshundahunsi, 2013; Garba & Kaur, 2014).

Groundnut protein concentrate (GPC) is extracted or prepared from groundnut through various methods such as isoelectric precipitation, aqueous precipitation, and alcoholic precipitation (Mouecoucou, Villaume, Sanchez, & Mejean, 2004). Several workers have reported the potential/benefits and the functional properties of GPC (Garba & Kaur, 2014; Gayol et al., 2013; Wu, Wang, Ma, & Ren, 2009; Yu, Ahmedna, & Goktepe, 2007). The objective of this study was to determine the functional and pasting properties of GPC and wheat flour blends. This will be useful in the development of new wheat‐based products in areas where wheat is highly consumed in different forms and protein deficiency is prevalent.

## 2 MATERIALS AND METHODS

### 2.1 Materials

Groundnuts (*Arachis hypogeal)* and wheat flour (Golden Penny) were obtained from Kure Central Market, Minna, Niger State.

### 2.2 Methods

#### 2.2.1 Preparation of defatted groundnut flour

Defatted groundnut flour was produced based on the procedure described by Kudre, Benjakul, and Kishimura (2013) with little modification. The nuts were sorted to remove extraneous materials and then pretreated for 5 min with a mixture of 5.25% sodium hypochlorite and de‐ionized water (1:10 v/v) to control microbial growth. Thereafter, the nuts were rinsed with de‐ionized water (1:3 w/v) and oven‐dried (NL9023A England) at 50°C for 24 hr. The nuts were then roasted at 140°C for 6 min, decoated manually and milled in a laboratory blender (Sa‐1706, China). Subsequently, the milled groundnut was mixed with chloroform/methanol (2:1 v/v) at a ratio of 1:10 (w/v). The mixture was heated at 60°C with continuous shaking (200 rpm) for 30 min in a water bath (NL42OS, England). It was then cooled to ambient temperature (32 ± 2°C) and centrifuged at 11,000 × *g* for 25 min at 15°C in an Avanti J‐E centrifuge (Beckman Coulter, Inc., Fullerton, CA, USA). The resulting pellet was air‐dried at ambient temperature (32±2°C) for 48 hr to rid it of solvent odor. It was then milled and packed in a plastic container (ZipLock, China) and stored in a cool place (10 ± 2°C) until required for use.

#### 2.2.2 Preparation of GPC

Groundnut protein concentrate was produced using the procedure reported by Gayol et al. (2013). Defatted groundnut flour was mixed with water at ratio 1/10 (w/v). Then it was shaken at ambient temperature for an hour, and the pH was modified to 4.5 with 4 mol/L concentration HCl. The suspension was centrifuged at 959 x g for 20 min. The supernatant was discarded, and the precipitate was resuspended in water at ratio 1/10 (w/v) and stirred at room temperature for 1 hr so as to clear the acid. Thereafter, it was centrifuged at 3,500 rpm for 30 min. The supernatant was discarded, and the precipitate was removed from the tube with a spatula, dried in an oven (Gallenkamp oven plus series) at 40°C, packaged, and stored at 4 ± 2°C until required for use.

#### 2.2.3 Preparation of blends of wheat flour and GPC

Wheat flour and GPC were blended (%, w/w) at ratios 100:0, 95:5, 90:10, 85:15, and 80:20, with 100% wheat flour serving as the control sample. The samples were homogenized with the aid of a Kenwood Mixer to obtain homogenous samples.

#### 2.2.4 Sample analyses

Protein, fat, ash, moisture, carbohydrate, fiber contents, and energy values were determined using standard methods (AOAC, 2000). Bulk density of the samples was determined using the method described by Kinsella (1981). Water absorption capacity was determined by the method described by Sosulski, Garrant, and Slinkard (1996). Emulsion capacity was determined using the method described by Yasumatsu et al. (1972). The foaming capacity was determined according to the method described by Narayana and Narsinga (1982). Swelling capacity and solubility index were determined according to procedures described by Akpata and Miachi (2001).

Pasting characteristics of blends were evaluated using a Brabender viscoamylograph (Newport Scientific Pty Ltd. Warrie‐wood NSW, Australia). Flour slurry, containing 12% solids, was heated from 30 to 95°C at a rate of 2.5°C/min, held at 95°C for 15 min, and cooled at the same rate to 50°C (Chinma, Gbadamosi, Ogunshina, Oloyede, & Salami, 2013). The pasting performance was automatically recorded on the graduated sheet of the amylogram. The peak viscosity, trough viscosity, breakdown viscosity, final viscosity, setback viscosity, peak time, and pasting temperature were read off the amylograph.

All experiments were performed in triplicate. Data obtained were subjected to one‐way analysis of variance while Duncan's multiple range test was conducted to separate the means. These were achieved using the Statistical Package for the Social Scientists (SPSS) version 23.0).

## 3 RESULTS AND DISCUSSION

### 3.1 Proximate composition of GPC

**Figure 1:**
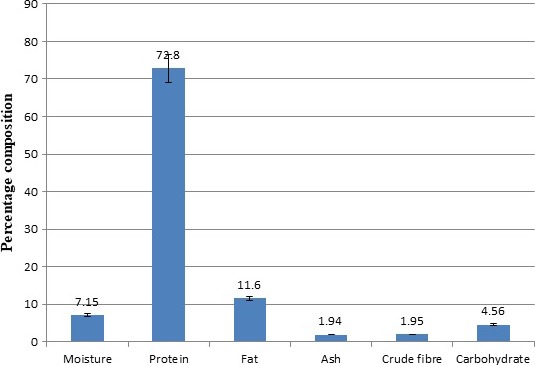
Proximate composition of groundnut protein concentrate

Proximate composition of GPC is presented in Figure 1. The protein content of the concentrate was 72.80%. This was lower compared with the average of 79.36% protein content obtained from groundnut flour at pH 8–10 (Gayol et al., 2013). The difference may probably be due to differences in extraction pH (Garba & Kaur, 2014). Fat and ash contents of 11.60% and 1.94%, respectively, were also lower than 17.00% and 4.85%, reported by Yu et al. (2007).

### 3.2 Effect of GPC inclusion on the proximate composition of wheat flour

Proximate composition of blends is presented in Table 1. Moisture content of the samples ranged from 8.0 to 9.0% with 100% wheat flour having the least value. Inclusion of GPC into wheat flour resulted in significant (*p* < .05) reduction in moisture content. This is advantageous because reduction in moisture content will reduce the proliferation of spoilage organisms especially mold, thus, improving the shelf stability of the product.

**Table 1:** Proximate composition (%) of wheat: GPC flour blends

Parameters	100:0	95:5	90:10	85:15	80:20
Moisture	9.1^a^ ± 0.3	8.03^b^ ± 0.02	8.50^b^ ± 0.10	8.00^b^ ± 0.01	8.10^b^ ± 0.10
Protein	14.70^e^ ± 0.10	18.40^d^ ± 0.01	21.56^c^ ± 0.11	25.26^b^ ± 0.10	28.87^a^ ± 0.25
Fat	1.93^e^ ± 0.13	5.56^d^ ± 0.15	6.88^c^ ± 0.30	7.37^b^ ± 0.18	8.84^a^ ± 0.18
Ash	0.70^c^ ± 0.05	1.05^b^ ± 0.01	0.95^b^ ± 0.06	0.96^b^ ± 0.04	1.13^a^ ± 0.08
Crude fiber	0.84^e^ ± 0.15	0.92^d^ ± 0.10	1.02^c^ ± 0.89	1.13^b^ ± 0.01	1.23^a^ ± 0.01
CHO	72.73^a^ ± 0.04	66.04^b^ ± 0.12	61.09^c^ ± 0.12	57.28^d^ ± 0.03	51.83^e^ ± 0.05

Values are means ± standard error of three determinations. Means in the same row with different superscript are significantly different (*p* < .05).

Significant (*p* < .05) increase in protein content of wheat flour was recorded with increasing GPC. Highest protein content of 28.87% was recorded in blend with 20% GPC. Vegetable proteins in forms of flour, concentrates, and isolates have been incorporated in many food systems for better nutritional values and functionality than products produced solely from wheat flour (Jideani, 2011; Olapade & Oluwole, 2013; Idowu, 2014). Fat, ash, and crude fiber contents also followed the same trend. These parameters ranged from 1.93% to 8.84%, 0.70% to 1.13%, and 0.84% to 1.23%, respectively. The increase may be due to the combination effect between wheat flour and groundnut protein concentrate. On the other hand, carbohydrate decreased significantly (*p* < .05) with increasing GPC. The increase in protein on the one hand, and the decrease in carbohydrate on the other hand, was a direct consequence of increasing and decreasing GPC and wheat flour, respectively.

### 3.3 Effect of GPC inclusion on the functional properties of wheat flour

The functional properties of a food material affect how it interacts with other food components and determines its application and end use. Therefore, food items with good functional properties can be easily incorporated into other foods and will yield good quality and acceptable end products. Functional properties of blends of wheat and groundnut concentrate flours are presented in Table 2. Bulk density ranged from 0.62 g/ml in sample containing 80% wheat flour and 20% GPC to 0.70 g/ml in 100% wheat flour. Bulk density decreased significantly (*p* < .05) with increasing GPC. Lower bulk density in the blends compared with 100% wheat flour could be a result of reduction in carbohydrate content which has been reported to have high bulk density (Gernah, Ariahu, & Ingbian, 2011). The density of flours is important as it affects mixing, packaging, and transportation. Nutritionally, low bulk density is advantageous because it engenders consumption of more quantity of the lighter food item and this will translate into more nutrients for the consumer.

**Table 2:** Functional characteristics of wheat: groundnut protein concentrate flour blends

Functional properties	100:0	95:5	90:10	85:15	80:20
Bulk density (g/ml)	0.70^a^ ± 0.01	0.68^b^ ± 0.01	0.66^b^ ± 0.01	0.68^b^ ± 0.01	0.67^b^ ± 0.01
Water absorption capacity (g/g)	0.90^c^ ± 0.01	0.90^c^ ± 0.01	0.93^b^ ± 0.21	1.13^a^ ± 0.01	1.14^a^ ± 0.01
Emulsification capacity (%)	27.58^c^ ± 0.98	32.10^b^ ± 0.01	32.10^b^ ± 0.01	37.04^a^ ± 0.01	37.04^a^ ± 0.01
Swelling capacity (%)	12.71^a^ ± 0.05	12.07^b^ ± 0.01	11.36^c^ ± 0.02	10.45^d^ ± 0.02	9.91^e^ ± 0.02
Foaming capacity (%)	5.25^d^ ± 0.25	5.90^c^ ± 0.14	5.85^c^ ± 0.35	8.10^b^ ± 0.14	9.20^a^ ± 0.02
Solubility index (%)	85.05^c^ ± 0.01	85.92^b^ ± 0.01	88.31^a^ ± 0.01	88.46^a^ ± 0.01	89.19^a^ ± 0.01

Values are means ± standard error of three determinations. Means in the same row with different superscript are significantly different (*p* < .05).

Inclusion of 5% GPC in wheat flour did not cause any significant (*p* ˃ .05) effect on water absorption capacity; however, there was a significant (*p* < .05) increase in water absorption capacity in blends with 10% groundnut concentrate and above. The increase in water absorption capacity could be due to high water absorption capacity of GPC which probably improved the structural matrix for holding water, sugars, and other components (Jideani, 2011). Jain, Prakash, and Radha (2015) reported a water holding capacity of 2.86 g/g for GPC.

Partial replacement of wheat flour with GPC resulted in significant (*p* < .05) increase in emulsion capacity. This may be due to the presence or increase in soluble proteins due to inclusion of GPC. According to Garba and Kaur (2014), soluble proteins enhance the emulsifying capacity of foods. This increase in emulsifying capacity indicated that inclusion of GPC in wheat flour will enhance the flours suitability for incorporation into food systems.

The foaming capacity increased significantly (*p* < .05) with increasing GPC in the blends probably due to the increased protein content of the blends. Brou et al. (2013) reported that foaming capacity is positively correlated with protein content.

Partial replacement of wheat flour with GPC at concentration of 10% and above resulted in significant (*p* < .05) increase in solubility index. This could be due to the high concentration of soluble protein in groundnut protein concentrate (Garba & Kaur, 2014). Jain et al. (2015) reported a solubility index ranging from 20% to >90% for GPC over a pH range of 2–10.

### 3.4 Effect of groundnut concentrate inclusion on the pasting properties of wheat flour

The pasting properties of a food refer to the changes that occur in the food as a result of application of heat in the presence of water. These changes affect texture, digestibility, and end use of the food product. Table 3 shows the effect of GPC incorporation on the pasting profile of wheat flour. Peak viscosity reduced significantly (*p* < .05) from 1,492 to 913 RVU with increasing GPC. Reduction in peak viscosity could be due to a lowering of the starch as well as interactions between the starch, fat, and protein contents of the blends. Peak viscosity has been reported to be correlated with water binding capacity of starch which takes place at equilibrium point between swelling which causes an increase in viscosity while rupturing and realignment cause its reduction (Sanni, Asiedu, & Ayernor, 2001). Peak time, trough, break, final, and setback viscosities also followed the same trend. Low breakdown viscosity exhibited by the blends is an indication of their ability to withstand breakdown during heating and shearing. According to Adebowale, Sanni, and Oladapo (2008), high breakdown viscosity could reduce the ability of flour to withstand heating and shear stress during cooking. There was no significant (*p* ˃ .05) difference between 100% wheat flour and blend containing 95% wheat flour and 5% GPC in pasting temperature. However, significant (*p* < .05) increase in pasting temperature was observed at concentration of 10% groundnut protein concentrate and above. Generally, the pasting properties (except pasting temperature) and the proximate composition of the flours indicate a direct relationship between the starch (carbohydrate) content and the pasting properties on the one hand, and an inverse relationship between protein and the pasting properties on the other hand. As the carbohydrate decreased, the pasting attributes also decreased, but as the protein increased, the pasting attributes decreased. The decrease in pasting attributes (except pasting temperature) with increasing protein may be due to the effect of protein on the starch. Chandrashekar and Kirleis (1988) observed that sorghum with higher protein content produced a thinner gruel and had a lower degree of starch gelatinization than sorghum with lower protein content and therefore inferred that proteins limit starch gelatinization. Furthermore, Derycke et al. (2005) reported that protein substantially affected the pasting properties of rice probably by reducing heat‐induced swelling of the starch. The reduction in the peak, trough, breakdown, set back, and final viscosities as well as the increase in pasting temperature of the flour blends with increasing protein content is similar to results reported by Ohizua et al. (2017) and Kiin‐Kabari, Eke‐Ejiofor, and Giami (2015). The higher pasting temperature observed with increasing GPC was probably due to the higher water absorption capacity observed in blends with higher GPC.

**Table 3:** Pasting properties of wheat: groundnut protein concentrate flour blends

Properties	100:0	95:5	90:10	85:15	80:20
Peak viscosity (RVU)	1,492^a^ ± 27	1,379^b^ ± 20	1,190.5^c^ ± 4.5	1,036^d^ ± 19	913^e^ ± 0.07
Trough viscosity (RVU)	820^a^ ± 21	759.5^a^ ± 30.5	675^b^ ± 4.4	608^c^ ± 11	536^d^ ± 0.00
Break down viscosity (RVU)	672^a^ ± 6	619.5^b^ ± 10.5	515^c^ ± 9	428^d^ ± 8	377^e^ ± 0.71
Final viscosity (RVU)	1,751^a^ ± 26	1,656^b^ ± 16	1,472.5^c^ ± 0.05	1,342^d^ ± 23	1,178^e^ ± 3.22
Set back viscosity (RVU)	931^a^ ± 5	896.5^b^ ± 5	797^c^ ± 4	734^d^ ± 12	642^e^ ± 3.43
Peak time (min)	6.00^a^ ± 0.67	5.83^b^ ± 0.01	5.80^c^ ± 0.67	5.67^c^ ± 0.00	5.57^c^ ± 0.03
Pasting temperature (°C)	88.03^b^ ± 0.03	88.00^b^ ± 0.05	89.25^a^ ± 0.35	89.58^a^ ± 0.04	90.03^a^ ± 0.43

Values are means ± standard error of three determinations. Means in the same row with different superscript are significantly different (*p* < .05).

## 4 CONCLUSION

This study showed the effect of the different levels of GPC in wheat flour on the proximate, functional, and pasting characteristics of the flour blends. The protein, fat, ash, and crude fiber contents of the blends improved with the inclusion of GPC while the moisture and carbohydrates reduced. Bulk density and swelling capacity of the flour blends reduced significantly (*p* < .05) with increasing level of GPC while solubility index, water absorption, emulsion, and foaming capacities increased. Peak time, peak, trough, breakdown, final, and setback viscosities of the flour blends reduced with GPC inclusion while the pasting temperature increased. Overall, wheat/groundnut protein concentrate flour blends showed good functional and pasting properties indicating that the blends can be used in the production of bread, cakes, biscuits, pancakes, and other baked products.

## CONFLICT OF INTEREST

None declared.

## ETHICAL STATEMENT

On behalf of all coauthors, I, Dr. Ocheme Boniface Ocheme, declare that this article has not been published in part or whole elsewhere. Neither is it under consideration for publication in another journal. All authors were actively involved in the work leading to the manuscript and will hold themselves jointly and individually responsible for its content.
